# Hyperbaric Oxygen Therapy Improves Parkinson’s Disease by Promoting Mitochondrial Biogenesis via the SIRT-1/PGC-1α Pathway

**DOI:** 10.3390/biom12050661

**Published:** 2022-04-30

**Authors:** Hung-Te Hsu, Ya-Lan Yang, Wan-Hsuan Chang, Wei-Yu Fang, Shu-Hung Huang, Shah-Hwa Chou, Yi-Ching Lo

**Affiliations:** 1 Faculty of Anesthesiology, School of Medicine, College of Medicine, Kaohsiung Medical University, Kaohsiung 80708, Taiwan; 2 Department of Anesthesia, Kaohsiung Medical University Chung-Ho Memorial Hospital, Kaohsiung 80756, Taiwan; 3 Department of Pharmacology, School of Medicine, College of Medicine, Kaohsiung Medical University, Kaohsiung 80708, Taiwan; tajen400221162@gmail.com (Y.-L.Y.); u109800003@kmu.edu.tw (W.-H.C.); tunafung@gmail.com (W.-Y.F.); 4 Graduate Institute of Medicine, College of Medicine, Kaohsiung Medical University, Kaohsiung 80708, Taiwan; huangsh63@gmail.com; 5 Division of Plastic Surgery, Department of Surgery, Kaohsiung Medical University Chung-Ho Memorial Hospital, Kaohsiung Medical University, Kaohsiung 80756, Taiwan; 6 Department of Surgery, School of Medicine, College of Medicine, Kaohsiung Medical University, Kaohsiung 80708, Taiwan; 7 Hyperbaric Oxygen Therapy Center, Kaohsiung Medical University Chung-Ho Memorial Hospital, Kaohsiung Medical University, Kaohsiung 80756, Taiwan; 8 Department of Medicine, Faculty of Medicine, College of Medicine, Kaohsiung Medical University, Kaohsiung 80708, Taiwan; shhwch@kmu.edu.tw; 9 Department of Chest Surgery, Kaohsiung Medical University Chung-Ho Memorial Hospital, Kaohsiung 80756, Taiwan; 10 Department of Medical Research, Kaohsiung Medical University Chung-Ho Memorial Hospital, Kaohsiung 80756, Taiwan; 11 School of Pharmacy, College of Pharmacy, Kaohsiung Medical University, Kaohsiung 80708, Taiwan

**Keywords:** Parkinson’s disease, hyperbaric oxygen therapy, mitochondrial biogenesis

## Abstract

Hyperbaric oxygen therapy (HBOT) has been suggested as a potential adjunctive therapy for Parkinson’s disease (PD). PD is a neurodegenerative disease characterized by the progressive loss of dopaminergic neurons in the substantia nigra pars compacta (SNpc). The aim of this study was to investigate the protective mechanisms of HBOT on neurons and motor function in a 1-Methyl-4-phenyl-1,2,3,6-tetrahydropyridine (MPTP) mouse model of PD and 1-methyl-4-phenylpyridinium (MPP^+^)-mediated neurotoxicity in SH-SY5Y cells on the potential protective capability. In vivo: male C57BL/6 mice were randomly divided into three groups: control, MPTP group and MPTP+HBOT group. The MPTP-treated mice were intraperitoneally received MPTP (20 mg/kg) four times at 2 h intervals within a day. The day after MPTP treatment, MPTP+HBOT mice were exposed to hyperbaric oxygen at 2.5 atmosphere absolute (ATA) with 100% oxygen for 1 h once daily for 7 consecutive days. In vitro: retinoic acid (RA)-differentiated SH-SY5Y cells were treated with MPP^+^ for 1 h followed by hyperbaric oxygen at 2.5 ATA with 100% oxygen for 1 h. The results showed that MPTP induced a significant loss in tyrosine hydroxylase (TH)-positive neurons in the SNpc of mice. HBOT treatment significantly increased the number of TH-positive neurons, with enhanced neurotrophic factor BDNF, decreased apoptotic signaling and attenuated inflammatory mediators in the midbrain of MPTP-treated mice. In addition, MPTP treatment decreased the locomotor activity and grip strength of mice, and these effects were shown to improve after HBOT treatment. Furthermore, MPTP decreased mitochondrial biogenesis signaling (SIRT-1, PGC-1α and TFAM), as well as mitochondrial marker VDAC expression, while HBOT treatment was shown to upregulate protein expression. In cell experiments, MPP^+^ reduced neurite length, while HBOT treatment attenuated neurite retraction. Conclusions: the effects of HBOT in MPTP-treated mice might come from promoting mitochondrial biogenesis, decreasing apoptotic signaling and attenuating inflammatory mediators in the midbrain, suggesting its potential benefits in PD treatment.

## 1. Introduction

Parkinson’s disease (PD) is a progressive, neurodegenerative, motor disorder, which affects ∼1% of the population aged 60 years and over [1]. PD is characterized by the degeneration of dopaminergic neurons within the substantia nigra pars compacta (SNpc), leading to symptoms of bradykinesia, resting tremor and muscle rigidity. The disease can also present with non-motor symptoms, such as sleep dysfunction, cognitive impairment and depression [2]. Although a variety of possible pathogenic mechanisms have been proposed over the years, including the excessive release of oxygen free radicals during enzymatic dopamine breakdown, damage to mitochondrial function, loss of nutritional support, and kinase activity abnormalities, destruction of calcium homeostasis and protein degradation dysfunction, detailed pathogenesis is still uncertain [3,4,5].

Hyperbaric oxygen therapy (HBOT) is a treatment that places patients in a hyperbaric chamber above 1.4 atmospheres absolute [6], allowing them to breathe pure oxygen naturally [7]. Compared with normal pressure, HBOT improves single-task or multi-task sports and cognitive performance in healthy humans [8]; however, the mechanism of this unique treatment is not yet fully understood. Research on the neuroprotective effects of HBOT have shown that hyperbaric oxygen can inhibit inflammation, reduce hypoxia and improve nervous system microcirculation [9]. There have been case reports of HBOT being used in the treatment of psychiatric symptoms in PD patients [10]. In addition, it has been shown in a Parkinson’s mouse model that HBOT can effectively inhibit the decrease in dopaminergic cells in the substantia nigra [11]. Although these research results show the potential of HBOT in the treatment of PD, the detailed neuroprotective mechanism remains unclear.

It is known that PD is associated with defects in mitochondrial respiration. This hypothesis arose in the late 1970s, when accidental exposure to 1-methyl-4-phenyl-1,2,3,6-tetrahydropyridine (MPTP), a contaminant from the synthesis of 1-methyl-4-phenyl-4-propionoxy-piperidine (MPPP), was found to cause parkinsonism and DA neurodegeneration [12]. There has also been strong recent evidence implicating mitochondrial dysfunction as a primary pathogenic pathway leading to the demise of dopaminergic neurons in PD [13]. The underlying mechanism may be the inhibition of complex I of the electron transport chain (ETC), which, in turn, increases reactive oxygen species [14,15]. The substantia nigra, compared to other brain regions, is more prone to mitochondrial complex I dysfunction, which results from the generation of ROS in the substantia nigra dopaminergic neurons during DA metabolism [16].

Therefore, mitochondrial function, particularly its preservation or promotion, is of special interest as a potential therapeutic target of PD and other neurodegenerative disorders. Mitochondrial biogenesis is a process by which new mitochondria are formed by the growth and division of preexisting mitochondria. It involves synthesis of the inner and outer mitochondrial membranes and mitochondrial-encoded proteins, synthesis and imports of nuclear-encoded mitochondrial proteins, and replication of mitochondrial DNA (mtDNA). Mitochondrial biogenesis is tightly regulated by several cell-signaling pathways. The sirtuin-1 (SIRT-1) and peroxisome proliferator-activated receptor gamma coactivator-1alpha (PGC-1α) is one such major pathway, and is considered to be the master regulator. PGC-1α is responsible for activating mitochondrial transcription factor A (TFAM) and binding to promoter regions of nuclear genes that encode subunits of the five complexes in the mitochondrial ETC, thereby increasing the assembly of the respiratory apparatus and regulating genes involved in heme biosynthesis, the import of nuclear-encoded mitochondrial proteins, and mtDNA replication and transcription [17].

For a long time, mitochondrial-targeting strategies have been studied for their potential in the treatment of brain injury and neurodegenerative diseases, in the hopes of providing neuroprotection by improving neuronal mitochondrial function. In different animal models of brain injury, HBOT has improved mitochondrial redox by enhancing mitochondrial function in neurons and glial cells to reduce oxidative stress, maintain mitochondrial integrity, and inhibit the mitochondrial-related apoptosis pathway to achieve a neuroprotective effect [18,19,20].

Since HBOT is non-invasive and is effective and safe for the treatment of many diseases, the purpose of this study was to use in vitro and in vivo models to investigate the detailed protective mechanisms of HBOT on the DA neurons of the SNpc, as well as to evaluate its therapeutic effects on motor symptoms in a Parkinson’s mouse model.

## 2. Materials and Methods

### 2.1. Reagents

First, 1-methyl-4-phenyl-1,2,3,6-tetrahydropyridine (MPTP) and 1-methyl-4-phenylpyridinium (MPP^+^) were purchased from Sigma-Aldrich (St. Louis, MO, USA). Tyrosine hydroxylase (TH) was purchased from Merck Millipore (Bedford, MA, USA). Brain-derived neurotrophic factor (BDNF), cyclooxygenase-2 (COX-2), B-cell lymphoma 2 (Bcl-2), Bcl-2-associated X protein (Bax), cytochrome c, mitochondrial transcription factor A (TFAM), and nuclear factor kappa B-p65 (NF-kB p65), were purchased from Santa Cruz Biotechnology (Santa Cruz, CA, USA). Phosphorylated cyclic AMP response element binding (pCREB), cyclic-AMP response element binding (CREB), sirtuin-1 (SIRT-1), peroxisome proliferator-activated receptor-γ coactivator 1-α (PGC-1α), cleaved caspase3, inducible nitric oxide synthase (iNOS), tumor necrosis factor alpha (TNF-α), and voltage-dependent anion channel (VDAC), were purchased from Cell Signaling Technology (Beverly, MA, USA). β-actin purchased from Abcam (Cambridge, MA, USA) and all other chemicals used in this study were of analytical grade.

### 2.2. Animals and MPTP-Treated Mice

C57BL/6 male mice (25–30 g) were used in this study. The mice were acclimatized for 7 days. The animals were maintained at (22 ± 2 °C) on 12:12-hour light/dark cycle and were allowed free access to pellet food and water. All experimental procedures conducted were approved by the institutional animal ethics committee. Eighteen male mice were randomly divided into 3 groups: Control group, MPTP group and MPTP+HBOT group (N=6 in each group). The MPTP group and MPTP+HBOT group mice were administered MPTP via intraperitoneal (*i.p.*) injection (20 mg/kg) four times at 2 h intervals within a day. The day after MPTP treatment, the MPTP+HBOT mice were exposed to hyperbaric oxygen at 2.5 ATA with 100% oxygen for 1 h in HBO treatment chamber (Genmall Biotechnology Co., Ltd., Taipei, Taiwan) once daily for 7 consecutive days [21]. At the end of the treatment schedule, behavioral tests were conducted. After behavior pattern analysis, the animals were anesthetized, sacrificed, and immediately dissected. Brain samples were obtained and Frozen at−80 °C for further analysis.

### 2.3. Locomotor Activity Test

The mice were placed in the center of the open-field test chamber (a white 50 × 50 × 25 cm chamber with an open top) and allowed to explore freely for 5 min. VideoTrack analysis system (ViewPoint Behavior Technology) was used to transmit the speed and distance of the experimental animals in a unit of time and analyzed with the VideoTrack software (Version 2.5.0.25, ViewPoint, Lyon, France).

### 2.4. Grip Strength Test

The grip strength of the fore and hind limbs of C57BL/6 mice was measured with a grip strength meter (Bioseb, Model: BIO-GS3, France). The mice were placed on the grip strength meter, and the mouse’s tail was pulled back at a constant speed after grasping the grid until it left the grid. The peak force in grams was recorded when the mice released the paws from the grid for each measurement. The analysis was performed using the mean peak force of ten trials. This test was performed by one person to ensure reliability.

### 2.5. Rotarod Performance Assessment

Rotarod was used to assess the motor coordination (Orchid Scientific, Maharashtra, India). The assessment depends upon the period of time that mice can retain themselves on a rotating rod. Prior to the test, each animal was given 1 min trial on the moving rod. They were placed on a rotating rod with acceleration ranges from 3 to 30 rpm and were assessed for their motor coordination for 300 s, and the latency to fall was recorded. Normal mice could retain themselves on the rotating rod for an indefinite duration of time. The motor performance was evaluated 3 times per day with 30 min intervals and the average retention time was calculated according to the previously studied protocol.

### 2.6. Immunohistochemistry

Substantia nigra tissues were fixed with 10% phosphate buffered formalin for 24 h, and paraffin sections were processed. The sections were then incubated with 0.3% H_2_O_2_ for 15 min at room temperature in the dark to exhaust endogenous peroxidase activity; then the slides were incubated with blocking buffer 3% bovine serum albumin (BSA) at room temperature for 30 min. After this, the tissue sections were incubated with primary antibody TH (1:500) at 4 °C overnight. Sections were washed three times in PBS then incubated with anti-rabbit horseradish peroxidase (HRP)-conjugated secondary antibody (1:400) for another 30 min at room temperature. The sections were developed with diaminobenzidine (DAB) and the images were observed under a light microscope.

### 2.7. Western Blot Analysis

In brief, isolated midbrain tissues were homogenized using ice-cold RIPA buffer containing protease inhibitor cocktail. The protein concentration was estimated by nanodrop spectrophotometer. First, 50 μg of protein was loaded onto the 10% SDS-PAGE and the separated proteins were transferred onto PVDF membranes. The membranes were blocked with 5% BSA for 1 h before being incubated with respective primary antibodies (1:1000) at 4 °C overnight. The membrane was incubated with secondary antibody HRP conjugate at room temperature for 60 min. Finally, each membrane was developed using an enhanced chemiluminescence method for the detection of HRP. Signals were quantified using “Image J” analysis software (version 1.53r or higher, U.S. National Institutes of Health, Bethesda, MD, USA).

### 2.8. SH-SY5Y Cell Culture

The SH-SY5Y human neuroblastoma cell line was purchased from ATCC (Manassas, VA, USA). Cells were cultured in SH-SY5Y growth medium consisting of Dulbecco’s Modified Eagle Medium (DMEM) supplemented with 10% fetal bovine serum (FBS) and 1% antibiotic antimycotic solution (Thermo Fisher Scientific, Waltham, MA, USA). In order to induce the differentiation of SH-SY5Y cells into dopaminergic cells, the SH-SY5Y cells were cultured for 3 days in MEM/F12 medium containing 10 μM retinoic acid and 3% FBS. Cells were maintained at 37 °C in a humidified atmosphere of 5% CO_2_.

### 2.9. Immunofluorescence Staining

The neurons were fixed in 4% paraformaldehyde, incubated in 0.2% Triton for 20 min to permeabilize the cells, and then blocked in 3% BSA for 40 min at room temperature. After being washed with PBS, the cells were then incubated with α-Tubulin primary antibodies at 4 °C overnight, and the bound antibodies were detected with Alexa Fluor 488 and Alexa Fluor 595 anti-rabbit IgG secondary antibody for 1 h at room temperature. After the cells were washed three times with PBS, the cell nuclei were then stained with DAPI for 10 min. The fluorescence images were visualized under fluorescence microscope (Carl Zeiss, Jena, Germany).

### 2.10. Statistical Analysis

The results are presented as the mean ± standard error of the mean (SEM) and were evaluated with one-way analysis of variance using Statistical Package for the Social Sciences (GraphPad Prism, software package version 5.0., San Diego, CA, USA.) and Tukey’s test was used to compare significant variation between the groups *p* < 0.05, *p* < 0.01 and *p* < 0.001.

## 3. Results

### 3.1. Hyperbaric Oxygen Therapy Attenuated Substantia Nigra Dopaminergic Neuronal Loss in MPTP-Treated Mice

**Figure 1:**
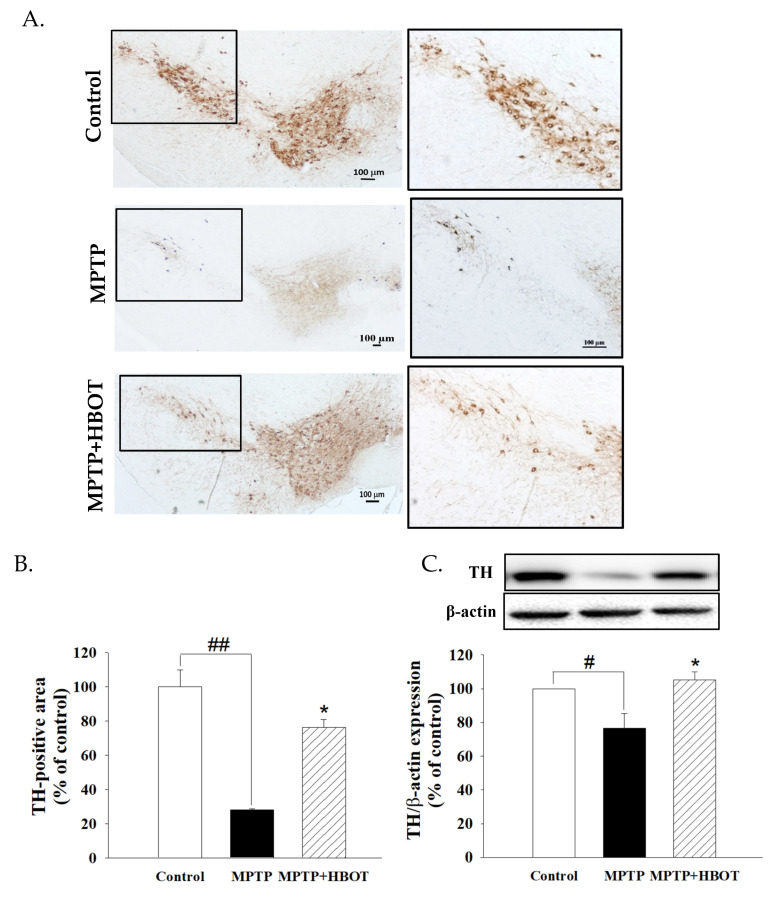
Effects of HBOT on TH-positive neurons in the SNpc of MPTP-treated mice. C57BL/6 mice were injected with MPTP (20 mg/kg, *i.p.*) four times at 2 h intervals for one day to induce PD model. One day after MPTP treatment, MPTP+HBOT group of mice were exposed to hyperbaric oxygen at 2.5 ATA with 100% oxygen for 1 h once daily for 7 consecutive days. (**A**) Representative photographs and (**B**) quantification of TH-positive neurons in SNpc. (**C**) Protein expression of TH in the mid-brain of MPTP-treated mice was detected by Western blotting. All data are expressed as mean ± SEM. (*n* = 5). # *p* < 0.05 and ## *p* < 0.01 compared with the control group; * *p* < 0.05 compared with the MPTP group.

PD is pathologically characterized by the death of dopaminergic neurons in the SNpc. We stained the tissues of the substantia nigra using a specific antibody against tyrosine hydroxylase (TH), which is a marker for dopaminergic neurons. As shown in Figure 1A,B, MPTP administration resulted in a drastic loss of dopaminergic neurons, showing a 28% loss of TH-positive neurons in the substantia nigra compared to the control (*p* < 0.01). HBOT showed a significant protective effect on dopaminergic neurons against MPTP-induced neurotoxicity, preserving up to 76% of TH-positive neurons compared to that of the MPTP group (*p* < 0.01). This was further confirmed by Western blot analysis, which demonstrated a higher TH protein level in the lesioned SNpc of HBOT-treated mice compared to that of the MPTP mice (Figure 1C). Therefore, HBOT attenuated the loss of dopaminergic neurons induced by MPTP in mice.

### 3.2. Hyperbaric Oxygen Therapy Improves Motor Activity and Grip Strength in MPTP-Treated Mice

**Figure 2:**
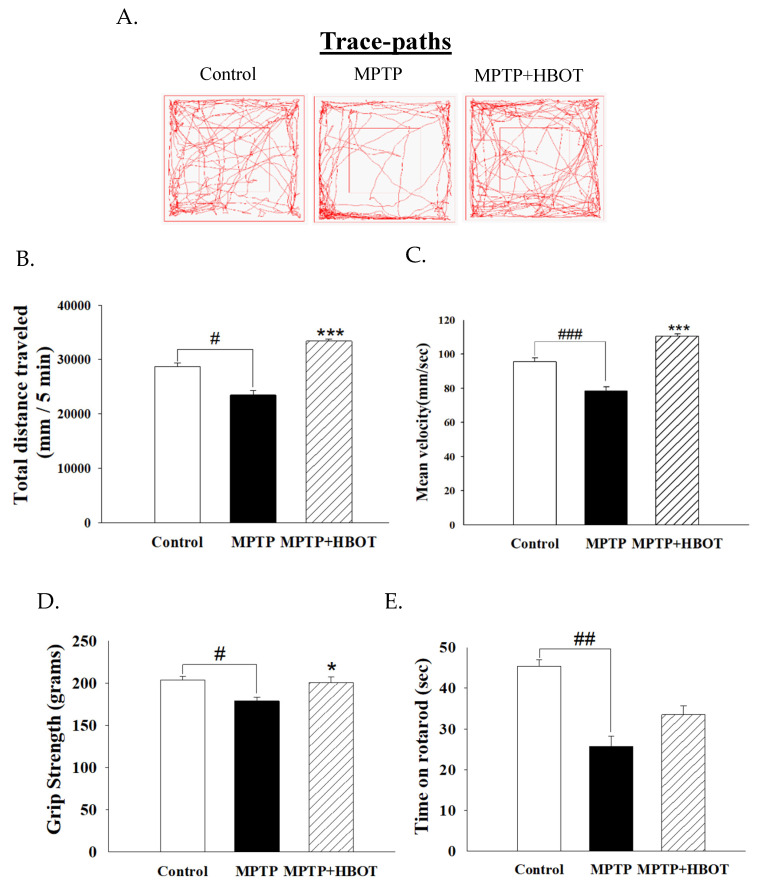
Effects of HBOT on locomotor activity, grip strength and motor performance in MPTP-treated mice. For locomotor activity test, (**A**) trace paths, (**B**) total distance and (**C**) mean velocity of mice were recorded in 5 min. (**D**) The maximum whole-limb muscle force was measured by grip strength meter. (**E**) Latency to fall was measured by rotarod. All data are expressed as mean ± SEM (*n* = 5). # *p* < 0.05, ## *p* < 0.01 and ### *p* < 0.001 compared with the control group; * *p* < 0.05 and *** *p* < 0.001 compared with the MPTP group.

To further explore the effect of HBOT on the motor symptoms of MPTP-treated mice, the locomotor activity test, grip strength test and rotarod test were administered. Following a 7-day treatment with HBOT, the total distance moved and mean velocity of the MPTP+HBOT treatment mice were significantly increased relative to the MPTP group (Figure 2B,C, *p* < 0.001 or *p* < 0.001, respectively). Furthermore, HBOT treatment enhanced extremity grip strength in MPTP+HBOT treatment mice to untreated MPTP mice (Figure 2D, *p* < 0.05). The results of the rotarod test are shown in Figure 2E, where the MPTP mice took significantly less time to fall off the rod than the control group (*p* < 0.01). Excitingly, the treatment of the MPTP mice with HBOT significantly increased the time taken to fall relative to untreated MPTP mice. These results indicate that HBOT improves the activity capability and muscle endurance of PD mice, and improves movement coordination, achieving the possible reversal of MPTP-induced Parkinsonian dyskinesia.

### 3.3. Hyperbaric Oxygen Therapy Inhibits Neuroinflammation in the Brain of MPTP-Treated Mice

**Figure 3:**
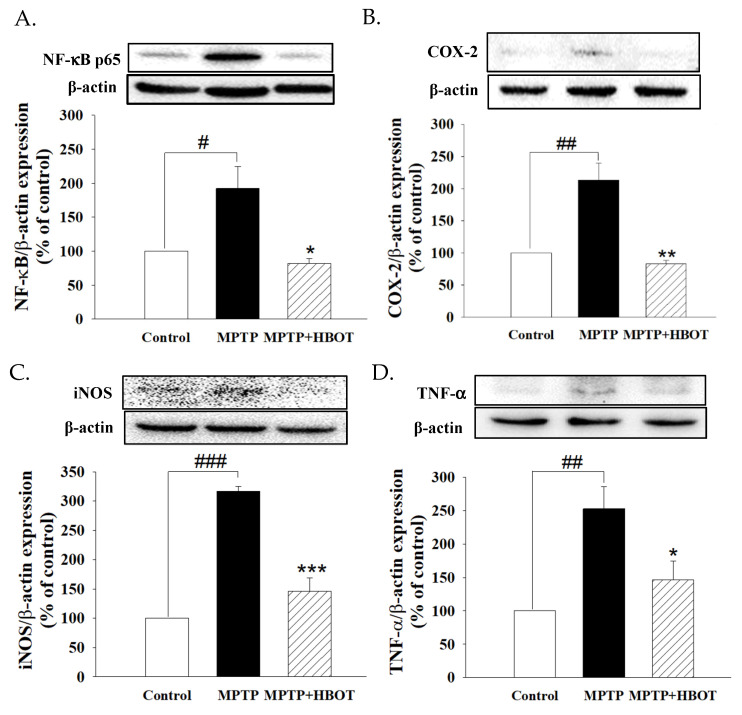
Effects of HBOT on the protein expression of inflammatory mediators in mid-brain tissue of MPTP-treated mice. Protein expression of (**A**) NF-κB p65, (**B**) COX-2, (**C**) iNOS and (**D**) TNF-α were detected by Western blotting. All data are expressed as mean ± SEM (*n* = 3). # *p* < 0.05, ## *p* < 0.01 and ### *p* < 0.001 compared with the control group; * *p* < 0.05, ** *p* < 0.01 and *** *p* < 0.001 compared with the MPTP group.

As shown in Figure 3, MPTP-treated mice showed significantly increased expression of NF-κB p65, COX-2, iNOS and TNF-α in brain tissue when compared to the control group (Figure 3A–D, *p* < 0.05, *p* < 0.01, *p* < 0.001 and *p* < 0.01, respectively). In contrast, mice treated with HBOT in MPTP-treated mice demonstrated drastically decreased levels of NF-κB p65, COX-2, iNOS and TNF-α expression compared to mice treated with MPTP alone (Figure 3A–D, *p* < 0.05, *p* < 0.01, *p* < 0.001 and *p* < 0.05, respectively).

### 3.4. Hyperbaric Oxygen Therapy Attenuates Apoptosis of Midbrain Tissue in MPTP-Treated Mice

**Figure 4:**
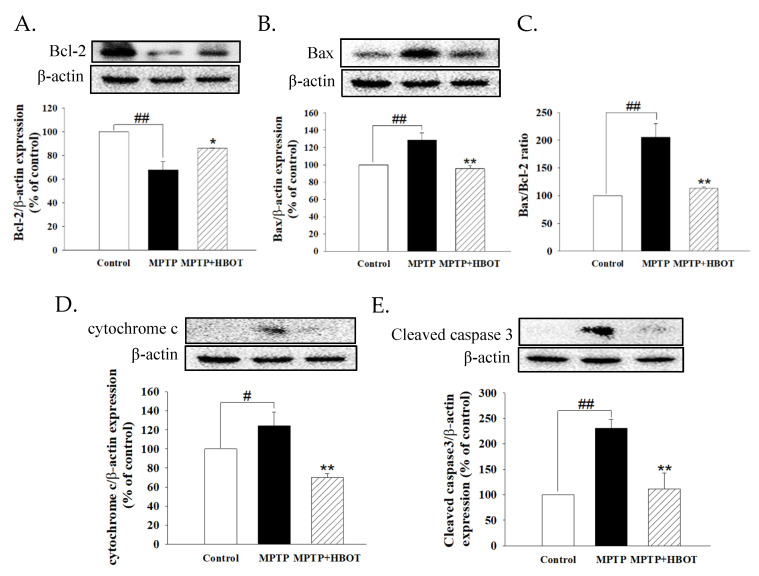
Effects of HBOT on the protein expressions of apoptotic pathway in the mid-brain tissue of MPTP-treated mice. Protein expression of (**A**) Bcl-2, (**B**) Bax, (**C**) Bax/Bcl-2 ratio, (**D**) cytochrome c, and (**E**) cleaved caspase 3 were detected by Western blotting. All data are expressed as mean ± SEM (*n* = 3–5). # *p* < 0.05 and ## *p* < 0.01 compared with the control group; * *p* < 0.05 and ** *p* < 0.01 compared with the MPTP group.

The expression of anti- and pro-apoptotic proteins Bcl-2 and Bax in MPTP-treated mice brain tissue were detected by Western blot. MPTP significantly decreased Bcl-2 but increased the expression of Bax, cytochrome c, and cleaved caspase 3 (Figure 4A,B,D,E, *p* < 0.01, *p* < 0.01, *p* < 0.05 and *p* < 0.01, respectively). HBOT could reduce these phenomena and decrease the ratio of Bax/Bcl-2 (Figure 4C, *p* < 0.01). These results indicate that HBOT attenuates the apoptosis of midbrain tissues in MPTP-treated mice.

### 3.5. Hyperbaric Oxygen Therapy Activates Neurotrophic Factor in MPTP-Treated Mice and Promotes the Neurite Outgrowth of MPP^+^-Treated SH-SY5Y Cells

**Figure 5:**
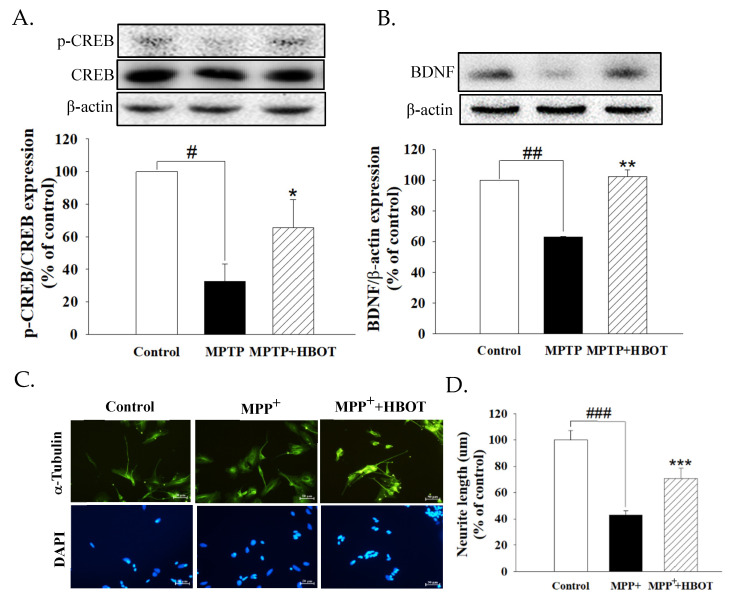
Effects of HBOT on the protein expressions of CREB/BDNF signaling pathway in the mid-brain tissue of MPTP-treated mice and the neurite outgrowth of MPP^+^-treated SH-SY5Y cells. Protein expression of (**A**) pCREB/CREB and (**B**) BDNF were detected by Western blotting. RA-differentiated SH-SY5Y cells were treated with MPP^+^ (1 mM) for 1 h followed by hyperbaric oxygen at 2.5 ATA with 100% oxygen for 1 h. (**C**) Cells were stained with anti-α-Tubulin (neuronal marker) antibody and counterstained with DAPI for observing neurite (scale bar = 50 µm) and (**D**) measuring the neurite length. All data are expressed as mean ± SEM (*n* = 3). # *p* < 0.05, ## *p* < 0.01 and ### *p* < 0.001 compared with the control group; * *p* < 0.05, ** *p* < 0.01 and *** *p* < 0.001 compared with the MPTP or MPP^+^ group.

We found that MPTP-treated mice showed significantly reduced expression of pCREB/CREB and BDNF in brain tissue when compared to the control group (Figure 5A,B, *p* < 0.05 and *p* < 0.01). HBOT treatment significantly increased the expression of pCREB/CREB and BDNF when compared with the MPTP mice (Figure 5A,B, *p* < 0.05 and *p* < 0.01, respectively). As shown in Figure 5D, the neurite length of SH-SY5Y cells was reduced by MPP^+^ (*p* < 0.001), whereas HBOT treatment attenuated neurite retraction (*p* < 0.001). These results indicate that HBOT promotes neurite outgrowth by activating the pCREB/BDNF pathway.

### 3.6. Hyperbaric Oxygen Therapy Promotes Mitochondrial Biogenesis through the SIRT-1/PGC-1α Pathway in MPTP-Treated Mice

**Figure 6:**
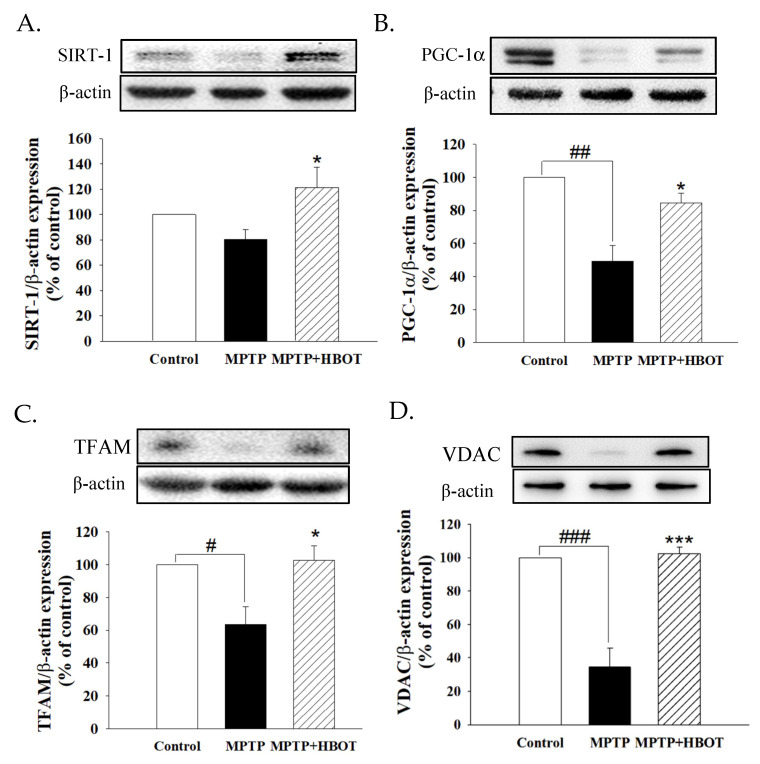
Effects of HBOT on the protein expressions of mitochondrial biogenesis in the midbrain tissue of MPTP-treated mice. Protein expression of (**A**) SIRT-1, (**B**) PGC-1α, (**C**) TFAM and (**D**) VDAC were detected by Western blotting. All data are expressed as mean ± SEM (*n* = 3–4). # *p* < 0.05, ## *p* < 0.01 and ### *p* < 0.001 compared with the control group; * *p* < 0.05 and *** *p* < 0.001 compared with the MPTP group.

In order to further investigate whether the neuroprotective mechanism of HBOT on MPTP-treated mice was related to mitochondrial functions, we performed Western blot to analyze mitochondrial-biogenesis-related proteins. As shown in Figure 6, MPTP-treated mice showed significantly reduced expression of SIRT-1, PGC-1α, TFAM and mitochondrial marker VDAC in brain tissue when compared to the control group (Figure 6A–D, *p* < 0.05, *p* < 0.01, *p* < 0.05 and *p* < 0.001, respectively). However, HBOT pretreatment in MPTP-treated mice increased the levels of SIRT-1, PGC-1α, TFAM and VDAC expression compared to the mice treated with MPTP alone. These results demonstrate that HBOT stimulates mitochondrial biogenesis in the midbrain of MPTP-treated mice via the SIRT-1/PGC-1α/TFAM pathway.

## 4. Discussion

In the present study, we examined the effects of HBOT treatment on locomotor activities and dopaminergic neurons in the substantia nigra of MPTP-treated mice. According to our results, locomotor activities, as quantified by the total distance traveled, grip strength, and the time on rotarod, were improved in the MPTP+HBOT mice (Figure 2).

Previous studies suggested that exposure to hyperbaric oxygen enhances the oxidative capacity of skeletal muscle fibers and the spinal motoneurons innervating them and, thus, has implications for locomotor activity. Our results appear to be concordant with these previous findings [22,23]. On the other hand, the selective degeneration of dopaminergic neurons and the reduced expression of TH in the SNpc, along with the decreased levels of DA transporter (DAT) and DA in the striatum, are the main pathological hallmarks of PD and correlate with motor dysfunction [24,25]. In our study, we observed that HBOT attenuated the decrease in dopaminergic cells and the reduced expression of TH in the midbrain of MPTP-treated mice (Figure 1). Therefore, HBOT appears to be a promising treatment for PD, with exciting clinical potential. In addition, there are also case reports of the use of HBOT in the treatment of psychiatric symptoms in PD patients [10]. Although our experiments did not examine the emotional effects of HBOT in the animal model of Parkinson’s disease, the potential interference of motoric impairment and locomotor expression in mood disorders are related to PD (anxiety, depression), which clearly demonstrated their impact on motor performance in the OF test [14,26]. Therefore, we cannot rule out the possibility that HBOT can improve the results of locomotor behaviors by improving the animal model’s psychiatric symptoms, and this requires further experiments to prove.

Although HBOT has an effect on the preservation of dopaminergic cells and motor function in MPTP-treated mice, the exact mechanism is still unclear. Neuroinflammation is known to be one of the most important processes involved in the pathogenesis of PD. MPTP is a potent neurotoxin used to mimic PD in a wide range of organisms, including non-human primates, guinea pigs, mice, dogs and cats [27]. Previous studies have demonstrated that mice treated with MPTP have significantly increased inflammatory mediators, such as iNOS, COX-2, and TNF-α in the SNpc [28,29]. In our study, we demonstrated that HBOT protects against MPTP-induced neuroinflammation in PD by inhibiting the NF-κB signaling pathway in the midbrain of MPTP-treated mice (Figure 3), indicating that HBOT has the effect of suppressing neuroinflammation.

In addition, the anti-apoptotic effect of HBOT has been demonstrated by mediating the enhancement of Bcl-2 expression and increasing intracellular oxygen bio-availability. Both of these may contribute to the preservation of mitochondrial integrity and reduce the activation of the mitochondrial pathway of apoptosis [19]. At the same time, HBOT also attenuates the decrease in the Bcl-2/Bax ratio, and reduces the expression of cytochrome c and the rising level of cleaved caspase 3 in the midbrain of MPTP-treated mice (Figure 4). These results indicate that HBOT has the anti-apoptotic effect to counter the apoptosis of DA neurons in PD.

Neurogenesis is defined as the generation of neurons within the brain. It has been suggested that HBOT exerts neuroprotective effects through the activation of cellular transcription factors [30]. The CREB is an intracellular protein that regulates the expression of genes that are important in dopaminergic neurons [31]. The activity of CREB in neurons has been correlated with various intracellular processes, including differentiation, survival, and neurogenesis [32]. Activated CREB promotes the expression of BDNF, which belongs to a family of neurotrophic that have a crucial role in neuronal survival and enhanced nerve transmission via long-term potentiation. This combination of neurogenesis and optimized neuronal function is believed to prevent neurogenerative diseases [33]. According to our results, HBOT may promote the activation of CREB and increase the expression of BDNF in the substantia nigra of MPTP-treated mice (Figure 5). The increase in neurite outgrowth is observed in the MPP^+^-treated SH-SY5Y cells with HBOT (Figure 5). The above results suggest that HBOT may promote neurogenesis via the activation of the CREB/BDNF pathway.

Mammalian sirtuins (SIRT-1–SIRT-7) have been implicated in a number of cellular and physiological processes, including gene silencing, apoptosis, mitochondrial function, energy homeostasis, and longevity [34]. Stimulation of SIRT-1 has been shown to increase the expression and activity of PGC-1α, a master regulator involved in mitochondrial biogenesis [35]. In cellular disease models, the activation of PGC-1α blocks dopaminergic neuron loss induced by mutant α-synuclein or the pesticide rotenone [36,37,38]. It acts as a coactivator for transcription factors, such as TFAM, which, in turn, increase mtDNA and mitochondrial biogenesis. In the substantia nigra of transgenic mice, inactivation of TFAM alleles induces respiratory chain deficiency in dopaminergic neurons and aggravates PD [39]. In addition, Voltage-Dependent Anion Channels (VDACs) also serve as docking sites for misfolded or mutated proteins, associated with many neurodegenerative disorders, including PD. Interactions with these abnormal proteins alter the physiological activity of VDAC, contributing to mitochondrial dysfunction typical of these pathologies [40,41]. The VDAC proteins represent the most important pore-forming proteins of the mitochondrial outer membrane, directly involved in metabolism and apoptosis regulation [42]. Therefore, the SIRT-1/PGC-1α signaling pathway plays a critical role in PD development and may be a potential target for PD therapy. In the present study, we demonstrated that HBOT caused a significant up-regulation of SIRT-1 expression, along with PGC-1α and its downstream transcription factor TFAM and mitochondrial marker VDAC (Figure 6). The increased mitochondrial biogenesis has been further confirmed by the increased expression of TH, the key enzyme in DA synthesis, in the brain tissue of MPTP+HBOT-treated mice. This also provided evidence for the hypothesis that HBOT treatment can improve mitochondrial biogenesis by activating the SIRT-1 dependent PGC-lα signaling cascade.

## 5. Conclusions

Currently, the cause of PD in humans remains unclear and despite continuous progress in research, most of the treatment options are symptomatic and non-curative. In the present study, we provide preliminary evidence that HBOT treatment may decrease neuroinflammation by inhibiting the NF-kB signaling pathway and promote neurogenesis via the activation of the CREB/BDNF pathway. Furthermore, this therapy may improve mitochondrial biogenesis via the SIRT-1/PGC-1α-dependent signaling cascade and have the ability to improve dopaminergic functions by increasing tyrosine hydroxylase expression. Hence, our results suggest that HBOT has potential as an adjunctive treatment, protecting dopaminergic neurons in the clinical treatment of PD.

## Data Availability

Data available upon request from the corresponding authors.
